# Health Tract, No. 86

**Published:** 1862-07

**Authors:** 


### HEALTH TRACT, No. 86.

### DIETING.

Some persons eat themselves to death, others are dieted to death. When a man is sick he is weak, and concludes that as when he was well he ate heartily and was strong, if he now eats heartily, he will become strong again; well-meaning, but ignorant friends are of the same opinion, and their solicitations to eat become one of the greatest annoyances of a sensible invalid. Nature purposely takes away the appetite under such circumstances, and makes the very sight of food nauseating. A sick man is feeble; this feebleness extends to every muscle of the body, and the stomach being made up of a number of muscles, has its share of debility. It requires several hours of labor for the stomach to "work up" an ordinary meal; and to give it that amount of work to do when it is already in an exhausted condition, is like giving a man, worn out by a hard day's work, a task which shall keep him laboring half the night. Mothers are often much afraid that their daughters will hurt themselves by a little work, if they complain of not feeling very well; and yet if such daughters were to sit down to dinner and shovel in enough provender for an elephant or a plowman, it would be considered a good omen and the harbinger of convalescence. A reverse procedure would restore multitudes of ailing persons to permanent good health; namely, to eat very little for a few days; eat nothing but coarse bread and ripe fruits, and work about the house industriously; or what is better, exercise in the open air for the greater part of each day on horseback, in the garden, or walking through the woodlands or over the hills, for hours at a time. Objectless walks and lazy lolling in carriages are very little better than nothing. The effect of interested, absorbing exercise, is to work out of the system the diseased and surplus matter which poisons it; this relieves the stomach of the burdens imposed upon it, and allows it time to gain strength, so as more perfectly to convert the food eaten into well-made, pure, and life-giving blood. A weakly but faithful servant, in the effort to get through with a specified amount of work, may perform it all, but none of it is thoroughly done; whereas, if a moderate task had been assigned, all of it would have been well done; so a weak stomach, indicated by a poor appetite, may be able to convert a small amount of food into pure, invigorating blood; but if too much is eaten, the attempt to "get through it all" is made, blood is manufactured, but it is an imperfect blood, it is vitiated, and mixing with that already in the system, at every beat of the heart, the whole mass is corrupted, and "I am ailing all over" is the expressive description. In another set of cases there is a morbid appetite; the unhappy dyspeptic is always hungry; and finding that he feels best while eating, and for a brief space afterward, he is always eating and always dying. To hear him talk, you would imagine he could not possibly live long, and yet he does live and grows old in his miseries. Such may reasonably expect a cure. 1st. By eating very moderately at three specified times each day, and not an atom at any other; then in less than a fortnight sometimes these distressing cravings will cease. 2d. Spend a large portion of daylight in agreeable out-door activities.

